# Gefitinib exposure and occurrence of interstitial lung disease in Japanese patients with non-small-cell lung cancer

**DOI:** 10.1007/s00280-019-03788-4

**Published:** 2019-02-14

**Authors:** Toshio Kawata, Mitsuo Higashimori, Yohji Itoh, Helen Tomkinson, Martin G. Johnson, Weifeng Tang, Fredrik Nyberg, Haiyi Jiang, Yusuke Tanigawara

**Affiliations:** 1 Clinical Pharmacology & Drug Safety and Metabolism Department, Science & Data Technology Division, R&D, AstraZeneca K.K., Osaka, Japan; 2 Statistics Group, Science & Data Technology Division, R&D, AstraZeneca K.K., Osaka, Japan; 3 Quantitative Clinical Pharmacology, Early Clinical Development, IMED Biotech Unit, AstraZeneca, Cambridge, UK; 4 Quantitative Clinical Pharmacology, Early Clinical Development, IMED Biotech Unit, AstraZeneca, Gaithersburg, MD USA; 5 Epidemiology, AstraZeneca R&D, Mölndal, Sweden; 6 Immuno-Oncology, Global Medicines Development, AstraZeneca R&D, Gaithersburg, MD USA; 7 Department of Clinical Pharmacokinetics and Pharmacodynamics, Keio University School of Medicine, 35 Shinanomachi, Shinjuku-ku, Tokyo, 160-8582 Japan

**Keywords:** Gefitinib, Population pharmacokinetics, Exposure-safety, Interstitial lung disease, α_1_-Acid glycoprotein

## Abstract

**Purpose:**

A prospective, multicenter, large-scale cohort with a nested case–control study (NCT00252759) was conducted to identify and quantify risk factors for interstitial lung disease (ILD) in Japanese patients with non-small-cell lung cancer who received gefitinib. This study reports the association between gefitinib exposure and the occurrence of ILD.

**Methods:**

A total of 1891 gefitinib plasma concentrations from 336 patients were measured after first dose, at steady state, and at time of ILD occurrence. Influences of demographic and pathophysiological factors on pharmacokinetics were investigated by non-linear mixed-effect modeling. The exposure to gefitinib was compared between patients without and with ILD occurrence to explore risks associated with gefitinib-induced ILD. Intra-patient comparison of exposure was also conducted between times at ILD development and normal states.

**Results:**

In the population pharmacokinetic analysis for gefitinib, α_1_-acid glycoprotein (AGP), age, body weight, and concomitant use of cytochrome P450 3A4 inducers were significant covariates on oral clearance (CL/F). AGP and body weight were also identified as factors affecting the volume of distribution. CL/F was significantly lower at the time of ILD occurrence than normal states. Patients who developed ILD tended to show higher exposure to gefitinib than those without ILD; however, these differences were not statistically significant. On the other hand, exposure at the time of ILD occurrence was significantly elevated compared to the time of normal state within the same patients.

**Conclusions:**

Significant elevation of exposure of gefitinib was observed at the time of ILD occurrence, suggesting reduction of CL/F could be associated with ILD-induced AGP elevation. Increase in exposure of gefitinib is unlikely to be a robust predictor of ILD and does not warrant any dose modifications.

**Electronic supplementary material:**

The online version of this article (10.1007/s00280-019-03788-4) contains supplementary material, which is available to authorized users.

## Introduction

Epidermal growth factor receptor (EGFR) tyrosine kinase inhibitors (TKIs) are a well-established therapy for the treatment of *EGFR*-activating mutation-positive non-small-cell lung cancer (NSCLC) [1]. EGFR TKIs are generally well-tolerated and are not associated with some of the side effects commonly reported with chemotherapeutic agents, such as anemia, neutropenia, and thrombocytopenia [2].

The EGFR TKI gefitinib (IRESSA^®^) was first approved for the treatment of advanced NSCLC in Japan in July 2002. In clinical trials and pre-approval clinical compassionate use, some reports of interstitial lung disease (ILD)-type events were observed. Following approval, gefitinib became widely available for clinical use and reports of ILD increased [3].

ILD is a disease that affects the parenchyma or alveolar region of the lungs [4]. When associated with drug use, it can present precipitously with acute, diffuse alveolar damage, which may be fatal in some patients [5]. ILD, especially idiopathic interstitial fibrosis, is a known comorbidity in patients with NSCLC and is associated with some lung cancer therapies [6]. It is also recognized that ILD is more common in Japanese populations compared with Western populations [6–9].

In collaboration with an academic team, AstraZeneca conducted a non-randomized cohort study with a nested case–control study to identify and quantify risk factors for ILD [3]. Statistical analysis was performed that identified risk factors for ILD occurrence, namely older age, poor World Health Organization (WHO) performance status, smoking history, recent NSCLC diagnosis, reduced lung coverage on computed tomography scan, pre-existing chronic ILD, and concurrent cardiac disease [3]. Investigation of proteomic biomarkers to identify proteins predictive of ILD identified 41 peptide peaks, representing 29 proteins [including α_1_-acid glycoprotein (AGP), α_1_-antichymotrypsin, α_2_-HS-glycoprotein, and haptoglobin], that predicted the development of ILD [10]. Whilst the association of drug-related adverse events with pharmacokinetic exposure to gefitinib has been reported previously [11–13], this has not been reported for ILD due to the low incidence of ILD in the population studies.

Here, we report the results from an exposure–response analysis of gefitinib for the occurrence of ILD in Japanese patients with NSCLC, based on population pharmacokinetic and multivariate logistic regression analyses.

## Materials and methods

### Study design

This observational study consisted of a case–control study of patients developing ILD and randomly selected control patients without ILD, nested within a defined non-randomized patient cohort (Online Resource, Supplementary Fig. 1). Patients were recruited from across 50 centers in Japan between November 12, 2003, and February 22, 2006. Patients with advanced/recurrent NSCLC who had received at least one chemotherapy regimen were enrolled into the cohort and were followed for up to 12 weeks after treatment initiation. Patients received either gefitinib (250 mg once daily) or chemotherapy, selected by the patient and treating physician. The study population included patients irrespective of whether their tumors were *EGFR* mutation positive or *EGFR* mutation negative. Patients who developed ILD in the cohort were registered into the case–control study as clinically diagnosed potential cases. For each potential case, four patients were randomly selected from patients in the cohort who had not yet developed ILD (“controls”, patients with advanced NSCLC who had received at least one chemotherapy regimen) (Online Resource, Supplementary Fig. 2). A Case Review Board of radiologists and clinicians subsequently confirmed the eligibility of all clinically diagnosed potential cases by blinded diagnostic review (“confirmed” cases).

### Ethics

All patients provided written informed consent. The study was done in accordance with the Declaration of Helsinki and International Conference on Harmonization and Good Clinical Practice guidelines. An ethics committee or institutional review board approved the final protocol at each study site.

### Subjects for analysis

#### Subject populations analyzed

All 336 patients who consented to pharmacokinetic assessment and provided measurable plasma concentrations of gefitinib were involved in the population pharmacokinetic analysis set. Of the 336 patients, a total of 51 were patients who developed ILD and were enrolled as “cases” in the case–control study. Of the remaining 285 patients, who did not develop ILD, 116 patients were enrolled as “controls” in the case–control study. The remaining 169 patients without ILD were not included in the case–control study but were included in the population pharmacokinetic analysis (Online Resource, Supplementary Fig. 1). Patient demographic information and subject characteristics for the population pharmacokinetic analysis are shown in Table 1.

**Table 1:** Demographic and other characteristics of the Japanese patients with NSCLC available for population pharmacokinetic and exposure-safety analysis

Variables	Value
Number of patients (*n*)	
With ILD	51
Without ILD	285
Sex (*n*)	
Male	184
Female	152
Age (years)	64.8 ± 9.1 (37–87)
Age group (*n*) (years)	
< 65	156
≥ 65	180
Height (cm)	159.4 ± 9.5 (130–182)
Body weight (kg)	55.5 ± 11.0 (33–100)
BMI (kg/m^2^)^a^	21.72 ± 3.40 (13.2–38.6)
Serum α_1_-acid glycoprotein (mg/dL)	121.3 ± 52.4 (31–327)
Aspartate aminotransferase (U/L)	24.7 ± 39.9 (9–720)
Alanine aminotransferase (U/L)	20.59 ± 38.79 (4.0–688.0)
Alkaline phosphatase (U/L)	347.1 ± 336.1 (85–3620)
Total bilirubin (mg/dL)	0.581 ± 0.742 (0.00–13.10)
Serum albumin (g/dL)	3.69 ± 0.55 (1.9–5.0)
Total protein (g/dL)	6.71 ± 0.70 (4.8–9.2)
Serum creatinine (mg/dL)	0.759 ± 0.206 (0.30–1.40)
Creatinine clearance (mL/min)^b^	75.02 ± 23.06 (26.5–151.6)
Concomitant use with CYP3A4 inducers (*n*)	
Yes	23
No	313
Concomitant use with CYP3A4 inhibitors (*n*)	
Yes	24
No	312
Concomitant use with PPIs and/or H_2_ antagonists (*n*)	
Yes	115
No	221

Data are mean ± standard deviation (range), unless otherwise stated

*BMI* body mass index, *CYP3A4* cytochrome P450 3A4, *ILD* interstitial lung disease, *PPI* proton pump inhibitor

^a^BMI was calculated according to Quetelet equation [14, 15]

^b^Creatinine clearance was calculated by Cockcroft–Gault equation [16]

#### Plasma concentrations of gefitinib and AGP

Six blood samples were taken from each patient at 1–3, 3–8, and 24 h after first treatment dosing (Day 1) and at steady state (Days 10–15). In addition, blood samples were taken from each patient with ILD, if possible, when ILD developed. Plasma concentrations of gefitinib were measured by a validated liquid chromatography/tandem mass spectrometry method with a lower limit of quantification of 0.5 ng/mL [17]. Gefitinib plasma concentrations were determined in 943 and 911 samples from 332 and 307 patients after first dosing and at steady state, respectively. Furthermore, 37 additional concentrations were determined at the time of ILD development from 27/51 patients with ILD. In total, data for 1891 plasma concentrations taken from 336 patients were used for population pharmacokinetic modeling.

When estimating the exposure at first dosing and at steady state, time-dependent pharmacokinetics were not assumed. Thus, both exposures at first dosing and steady state can be theoretically derived if at least one plasma concentration at either first dosing or steady state is available. Individual exposures were predicted based on patients’ dosing history, assuming steady state at 144 h after the first dosing. As a result, predicted exposure at first dosing was obtained from all 336 patients, and that at steady state from 310 patients who were still receiving gefitinib after 144 h. Serum concentration of AGP were likewise determined at first dosing and at steady state.

### Population pharmacokinetic model development

The population pharmacokinetic analysis was performed using the non-linear mixed-effect modeling (NONMEM) program version V level 1.1 with the PREDPP library and the NM-TRAN pre-processor (GloboMax LLC, Hanover, MD, USA) [18]. Full details of the population pharmacokinetic model development and analysis are described in the Online Resource.

### ILD incidence versus exposure quartiles

Area under the plasma concentration–time curve from 0 to 24 h (AUC_0–24_) and maximum plasma concentrations (*C*_max_) were calculated using empirical Bayesian estimates of pharmacokinetic parameters in the final population pharmacokinetic model. AUC_0–24_ and *C*_max_ were chosen as exposure metrics. The incidence of ILD was summarized for four exposure levels, defined using exposure quartiles after a single dose and at steady state, respectively.

### Association between exposures to gefitinib and incidence of ILD

In patients with samples that were available for pharmacokinetic assessment, the association between the development of ILD and exposure to gefitinib was explored by inter-patient comparison of gefitinib exposure between patients with/without ILD using box plots and the paired *t* tests. Although plasma concentrations at the time of ILD development were available in 27 of 51 patients with ILD, only 19 of those 27 patients were used for the intra-patient comparison, as they had data available for exposure at the time of ILD development, and at steady state other than ILD development; the remaining 8 of the 27 patients discontinued gefitinib treatment prior to pharmacokinetic assessment at steady state and only had baseline measurements available. The remaining 24 of the 51 ILD cases had no sample at the time of ILD development.

### Association of ILD with gefitinib exposure and other risk factors for ILD using a multivariate logistic regression model

Patients with and without ILD, in whom pharmacokinetic measurements were available, were included in this analysis. The rationale for including both sets of patients was to identify risk factors which were associated with gefitinib exposure in patients with ILD. A multivariate logistic regression model was used in this analysis; the analysis was performed using SAS version 9.3.

Based on a preliminary exploratory analysis and the previous results of the full study analysis [3], age (< 55, ≥ 55 years), WHO performance status (0, 1, 2–3), smoking history (smoker versus never-smoker), duration of NSCLC (< 0.5, 0.5–1, > 1 year), severity of pre-existing ILD (no, mild, moderate to severe), severity of pre-existing pulmonary emphysema (no, mild, moderate, severe), and normal lung coverage (10–50%, 60–100%) were investigated as risk factors [3]. In addition, the interaction of smoking history and normal lung coverage, and the interaction of the severity of pre-existing ILD and normal lung coverage were also studied. In addition, pharmacokinetic exposure parameters were divided into four quartiles (see “Results”) and investigated as risk factors. For association with ILD risk, the mid-1, mid-2, and high-exposure groups were compared with the low-exposure group.

## Results

### Population pharmacokinetics of gefitinib

The population pharmacokinetics of gefitinib were described based on a one-compartment model, after first-order absorption with lag time (Table 2). In our model, statistically significant covariates were AGP, age group, body weight, and concomitant use of cytochrome P450 3A4 (CYP3A4) inducers for oral clearance (CL/F), and AGP and body weight for volume of distribution (V/F). In addition, CL/F was significantly lower at the time of ILD diagnosis. As shown in the Online Resource (Supplementary Figs. 3 and 4), the final model showed good agreement between the observed and predicted concentrations and was able to reliably predict steady state plasma concentrations of gefitinib. The obtained parameter estimates for gefitinib are summarized in Table 2. According to the estimated parameters, CL/F and V/F increased with an increase in body weight, and the exponents of AGP terms on the CL/F and V/F equations were negative values, suggesting that an increase in AGP induced a decrease in CL/F and V/F. Furthermore, it was found that CL/F declined in elderly patients aged ≥ 65 years, and increased in patients using CYP3A4 inducers.

**Table 2:** Estimated population pharmacokinetic parameters for gefitinib in patients with NSCLC

Parameters	Estimates	95% bootstrap confidence intervals
$${\text{CL/}}F~({\text{L/h}})=~{\theta _{1~}} \times {\left( {\frac{{{\text{AGP}}}}{{121}}} \right)^{{\theta _2}}}~ \times ~\theta _{3}^{{~{\text{AGE}} \geq 65}}~ \times ~\theta _{4}^{{{\text{ IND}}}}~ \times ~{\left( {\frac{{{\text{WGT}}}}{{55.5}}} \right)^{{\theta _5}}} \times ~\theta _{6}^{{{\text{ PERI}}}}$$		
*θ*_1_	28.6	(26.7, 30.4)
*θ*_2_	− 0.502	(− 0.561, − 0.445)
*θ*_3_	0.759	(0.676, 0.853)
*θ*_4_	1.82	(1.49, 2.10)
*θ*_5_	0.503	(0.273, 0.757)
*θ*_6_	0.743	(0.673, 0.850)
$$V{\text{/}}F~({\text{L}})=~{\theta _7} \times {\left( {\frac{{{\text{AGP}}}}{{121}}} \right)^{{\theta _{\text{8}}}}}~ \times ~{\left( {\frac{{{\text{WGT}}}}{{55.5}}} \right)^{{\theta _9}}}$$		
*θ*_7_	1540	(1490, 1620)
*θ*_8_	− 0.447	(− 0.519, − 0.377)
*θ*_9_	0.766	(0.511, 1.05)
*k*_a_ (h^−1^) *= θ*_10_	0.819	(0.749, 0.900)
*t*_lag_ (h) *= θ*_11_	0.863	(0.858, 0.954)
*ω*_CL/F_ (%)	49.3 (− 4.6%)^a^	(45.6, 52.0)
*ω*_V/F_ (%)	38.5 (7.1%)^a^	(31.3, 44.4)
${\omega _{{k_{\text{a}}}}}$ (%)	266 (57.0%)^a^	(250, 282)
${\omega _{{t_{{\text{lag}}}}}}$ (%)	26.5 (0.6%)^a^	(7.15, 33.8)
*σ* (%)	32.7 (31.2%)^b^	(31.0, 34.1)

All covariates were statistically significant at the *p* < 0.001 level

AGE ≥ 65 is 0 for patients aged < 65 years and 1 for patients aged ≥ 65 years

IND is 0 for no concomitant use with CYP3A4 inducers and 1 for concomitant use with CYP3A4 inducers

PERI is 0 at the time of no ILD diagnosis and 1 at the time of ILD diagnosis

*AGP* serum α_1_-acid glycoprotein (mg/dL), *CL*/*F* oral clearance, *CYP3A4* cytochrome P450 3A4, *ILD* interstitial lung disease, *NSCLC* non-small-cell lung cancer, *V*/*F* volume of distribution, *WGT* body weight (kg)

^a^η-shrinkage

^b^ε-shrinkage

### Association between exposure to gefitinib and development of ILD

**Fig. 1:**
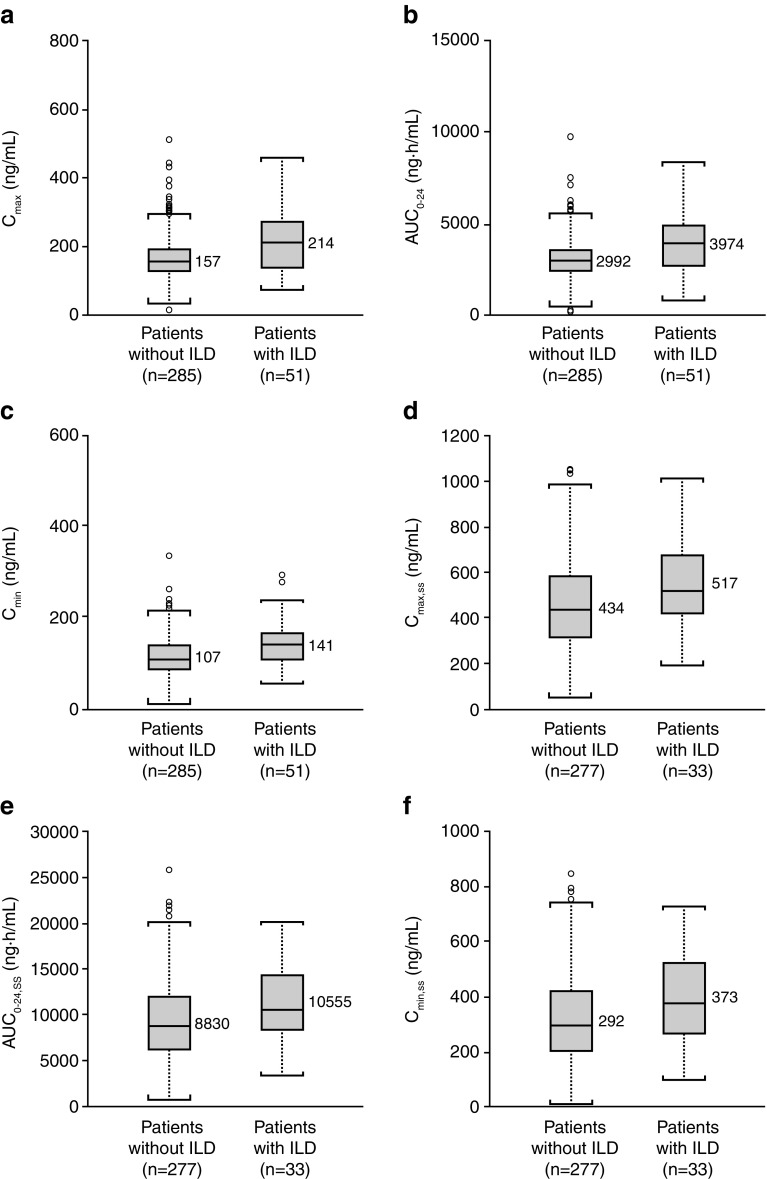
Inter-patient comparison of exposure of gefitinib between patients with NSCLC who later developed and who did not develop ILD. Box plots show the predictability of future ILD development: **a** by *C*_max_ after the first dosing; **b** by AUC_0–24_ after the first dosing; **c** by *C*_min_ after the first dosing; **d** by *C*_max,ss_ at steady state; **e** by AUC_0–24,ss_ at steady state; and **f** by *C*_min,ss_ at steady state. The middle line within the box indicates the median value of the data. The upper and lower edges (hinges) of the box indicate the first and third quantiles of the data, respectively. The end caps (staples) of the vertical dotted lines “whiskers” show the minimum or maximum data values, unless outliers are present, in which case the whiskers extend to a maximum of 1.5 times the inter-quartile range. The open circles outside the end caps of the whiskers indicate outliers or suspected outliers. *AUC*_*0–24*_ area under the plasma concentration–time curve from 0 to 24 h after a single dose, *AUC*_*0–24,ss*_ area under the plasma concentration–time curve from 0 to 24 h at steady state, *C*_*max*_ maximum plasma concentration after a single dose, *C*_*max,ss*_ maximum plasma concentration at steady state, *C*_*min*_ minimum plasma concentration after a single dose, *C*_*min,ss*_ minimum plasma concentration at steady state, *NSCLC* non-small cell lung cancer;* ILD* interstitial lung disease

**Fig. 2:**
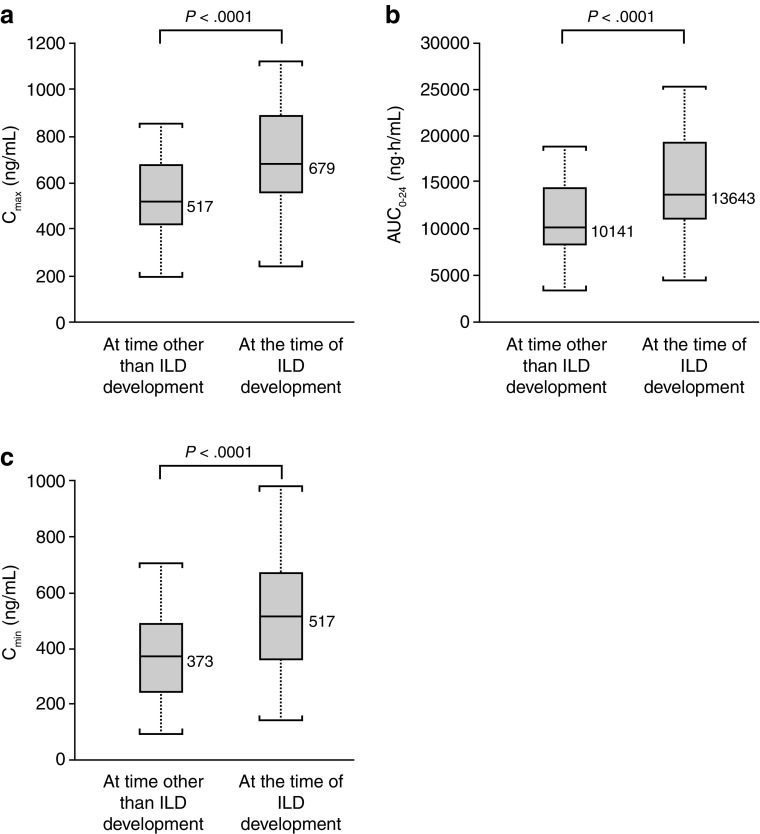
Intra-patient comparison of exposure of gefitinib at a time point before ILD development with those at the time of ILD development. Comparisons were performed in the cases (*n* = 19) in which exposure of gefitinib was available at both steady state without ILD and at the ILD occurrence. Box plots show: **a** the association between *C*_max_ and ILD development; **b** the association between AUC_0–24_ and ILD development; and **c** the association between *C*_min_ and ILD development. ILD represents “at the time of ILD development” and non-ILD represents “at time other than ILD development”. The middle line within the box indicates median value of the data. The upper and lower edges (hinges) of the box indicate the first and third quantiles of the data, respectively. The end caps (staples) of the vertical dotted lines “whiskers” show the minimum or maximum data values, unless outliers are present in which case the whiskers extend to a maximum of 1.5 times the inter-quartile range. The open circles outside the end caps of the whiskers indicate outliers or suspected outliers. *AUC*_*0–24*_ area under the plasma concentration–time curve from 0 to 24 h after a single dose, *C*_*max*_ maximum plasma concentration after a single dose, *C*_*min*_ minimum plasma concentration after a single dose, *ILD* interstitial lung disease. *P* values were derived from paired *t* test

Predicted exposures [AUC_0–24_, *C*_max_, and minimum plasma concentration (*C*_min_)] of gefitinib at first dosing in patients who developed ILD tended to be higher than in those of patients without ILD (Fig. 1a–c). A similar pattern was observed in exposure to gefitinib at steady state (Fig. 1d–f). Comparing exposures to gefitinib within 19 patients, from whom both steady state exposures at time other than ILD development and at the time of ILD development were available, showed that all exposure parameters were significantly elevated (at the 5% significance level) at the time of ILD development (Fig. 2) in comparison with earlier measured steady state in the same patient.

### Incidence of ILD versus exposure quartiles

After a single dose, predicted pharmacokinetic values were available for all 51 patients with ILD and all 285 patients without ILD. These data were used to calculate the quartiles of AUC_0–24_ and *C*_max_ after a single dose. At steady state, due to withdrawal or other clinical reasons, the predicted pharmacokinetic values were only available for 33 patients with ILD and 277 patients without ILD, and these data were used to calculate the quartiles of AUC_0–24,ss_ and *C*_max,ss_ at steady state. Each pharmacokinetic parameter was quartiled separately. There appeared to be a higher incidence of ILD in the highest exposure quartile for pharmacokinetic parameters after a single dose (AUC_0–24_ and *C*_max_), but this tendency was less clear for pharmacokinetic parameters at steady state (AUC_0–24,ss_ and *C*_max,ss_) (Table 3).

**Table 3:** Summary of the proportion of study patients with ILD in the study sample, by quartiles of gefitinib exposure (after a single dose and at steady state)

Quartile	No. patients in quartile, *n*	AUC^a^		*C*_max_^b^	
		No. cases	Proportion (%)	No. cases	Proportion (%)
After a single dose of gefitinib					
Quartile 1	84	10	11.9	10	11.9
Quartile 2	84	7	8.3	7	8.3
Quartile 3	84	7	8.3	8	9.5
Quartile 4	84	27	32.1	26	31.0
Total	336	–	–	–	–
At steady state					
Quartile 1	78	4	5.1	5	6.4
Quartile 2	77	6	7.8	5	6.5
Quartile 3	78	12	15.4	14	17.9
Quartile 4	77	11	14.3	9	11.7
Total	310	–	–	–	–

*AUC*_*0–24*_ area under the plasma concentration–time curve from 0 to 24 h after a single dose, *AUC*_*0–24,ss*_ area under the plasma concentration–time curve from 0 to 24 h at steady state, *C*_*max*_ maximum plasma concentration after a single dose, *C*_*max,ss*_ maximum plasma concentration at steady state, *ILD* interstitial lung disease, *Q* quartile

^a^AUC is: AUC_0–24_ for single dose data; and AUC_0–24,ss_ for steady state data

^b^*C*_max_ is: *C*_max_ for single dose data; and *C*_max,ss_ for steady state data

### Association of ILD with exposure to gefitinib and other risk factors for ILD using a multivariate logistic regression model

**Fig. 3:**
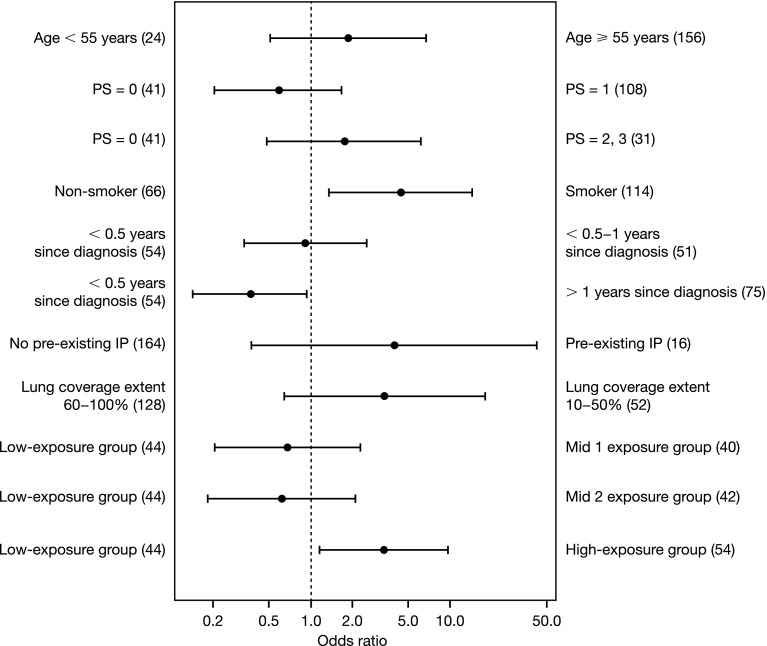
Odds ratios and 95% confidence intervals for ILD in Japanese patients with NSCLC associated with AUC_0–24_ after a single dose and other risk factors for acute ILD, from the final logistic regression model. The number of records in each subgroup is presented in parentheses. The number of records used for the logistic model analysis was 180 for single dose variables. An odds ratio > 1 indicates that a subgroup on the right hand side has a greater risk of ILD than the reference subgroup on the left hand side. *AUC*_*0–24*_ area under the plasma concentration–time curve from 0 to 24 h after a single dose, *ILD* interstitial lung disease, *IP* interstitial pneumonia, *NSCLC* non-small-cell lung cancer, *PS* performance status

Data used for the logistic regression analysis with pharmacokinetic variables consisted of patients with and without ILD treated with gefitinib. The odds ratios estimated using logistic regression analysis relating the association of ILD to different risk factors and gefitinib exposure parameters after a single dose (AUC_0–24_ and *C*_max_) and at steady state (AUC_0–24,ss_ and *C*_max,ss_) are presented in the Online Resource (Supplementary Table 1), and the odds ratios for the association of ILD with AUC_0–24_ after a single dose and other risk factors for ILD are also presented graphically in Fig. 3.

Patients in the high-exposure group when compared with the low-exposure group had odds ratios significantly greater than 1 for both pharmacokinetic exposure parameters after a single gefitinib dose (AUC_0–24_ and *C*_max_), although these were only slightly lower than the odds ratios for smoking history and normal lung coverage (Online Resource, Supplementary Table 1). These data indicate that the association of these factors with ILD incidence was significant and/or important. The results showed that patients with AUC and *C*_max_ in the high-exposure group after single dose administration exhibited a higher risk of ILD compared with the low-exposure group. Moreover, patients with pharmacokinetic exposures lower than or equal to the third quartile (i.e., an AUC_0–24_ ≤ 3855 ng·h/mL or a *C*_max_ ≤ 207 ng/mL) appeared to have a lower risk for ILD than those in the fourth quartile [odds ratios for fourth quartile versus first–third quartile (95% confidence intervals): 4.42 (1.85, 10.59) for AUC_0–24_, and 4.76 (1.98, 11.47) for *C*_max_]. These odds ratios were obtained using the same logistic regression model and covariates but with the respective pharmacokinetic variables dichotomized at the third quartile instead. Pharmacokinetic exposure parameters at steady state (AUC_0–24,ss_ and *C*_max,ss_) did not show any statistically significant association with ILD incidence in the logistic regression models (Online Resource, Supplementary Table 1). At steady state conditions, the association of ILD incidence with normal lung coverage (10–50%) or smoking history was statistically significant.

## Discussion

In the circulation, gefitinib is predominantly bound to human plasma protein and binding to human AGP ranges from 68.5% at 8 mg/mL to 83.0% at 0.05 mg/mL [19]. Therefore, the inter-individual variability of AGP may have an effect on the plasma kinetics of gefitinib. In fact, our population pharmacokinetic model described CL/F and V/F to be significantly decreased with increased AGP; thus, AGP is an important factor in defining the pharmacokinetic profile of gefitinib. In vitro and in vivo studies have also demonstrated that CYP3A4 is the major enzyme involved in the metabolic clearance of gefitinib [20]; therefore, it is rational that our data described CL/F increases in patients receiving CYP3A4 inducers. In a previous interaction study with rifampicin, which is a known strong CYP3A4 inducer [21], the AUC of gefitinib was decreased by approximately 83% in the presence of rifampicin, and a similar influence was observed in our population pharmacokinetic analysis. However, concomitant use of CYP3A4 inhibitors and proton pump inhibitors/H_2_ antagonists was not identified as a significant covariate in our population pharmacokinetic analysis, suggesting these data are inconsistent with the previous clinical observations. An interaction study with itraconazole, a strong CYP3A inhibitor, reported the AUCs of gefitinib 250 mg and 500 mg to be increased by 78% and 61%, respectively, in the presence of itraconazole [21]. Finally, a clinical pharmacology study to assess the effect of an increase in gastric pH on the relative bioavailability of gefitinib demonstrated that a sustained elevation of gastric pH decreased the bioavailability of gefitinib by 47% [22]. In this study, patients receiving CYP3A4 inducers, CYP3A4 inhibitors, proton pump inhibitors, or H_2_ antagonists during days when a concomitant use may affect pharmacokinetic evaluation of gefitinib were identified, but their detailed dosing histories of concomitant drug were not collected. Thus, the drug–drug interactions reported in the above publications were not confirmed in this analysis. Furthermore, detecting the effect of CYP3A4 inhibitors or proton pump inhibitors/H_2_ antagonists on the exposure of gefitinib could prove challenging due to the potential time difference between blood samples being taken and the time when any concomitant drugs were present in the circulation.

Hirose et al. reported that a patient who died of gefitinib-related ILD had their highest AUC_0–24_ and *C*_max_ on Day 1 after treatment with gefitinib, and suggested that high exposure to gefitinib may be associated with ILD development [11]. In our analysis, higher exposure to gefitinib at single dose and steady state was observed in patients who subsequently developed ILD than those who did not, and there appeared to be a higher incidence of ILD at the highest exposure (Table 3). In the population pharmacokinetic analysis, CL/F of gefitinib was decreased by 25.7% at the time of ILD development, resulting in an increase in exposure of gefitinib at time of ILD development (Fig. 2). Data supporting these findings include a patient case study which reported that the trough plasma concentration of erlotinib was elevated at the time of ILD diagnosis [23], and a report that median trough concentrations of erlotinib in three patients at the time of suspected ILD were approximately three times higher than those in patients without ILD [24]. Emoto-Yamamoto et al. also speculated that these findings might be the result of a decrease in erlotinib CL/F caused by ILD-induced AGP elevation [25]. Unfortunately, AGP measurements at the time of ILD development were not performed in our cohort and the nested case–control study. However, considering a report that AGP increases at the time of ILD development [26], a decrease in gefitinib CL/F at the time of ILD occurrence may be caused by an elevation of AGP. Since our population pharmacokinetic model showed AGP was a significant covariate decreasing gefitinib CL/F, pharmacokinetic property at the time of ILD occurrence would be described by AGP, if AGP at the time of ILD occurrence was determined. This is consistent with an investigation of proteomic biomarkers in our nested case–control study, which suggested that analysis of AGP could be used to predict ILD [10]. It must be noted that our population pharmacokinetic model described a decrease in gefitinib CL/F if AGP escalated, as was the case after ILD development. Thus, increased gefitinib exposure observed at the time of ILD development could be a result of ILD-induced AGP elevation and higher exposure may therefore be a result of, rather than a cause of, developing ILD. Smoking history and normal lung coverage (< 50%) were also determined as significant risk factors of ILD occurrence even in this smaller subsample of patients, consistent with previous results from the full case–control data of the study [3]. It must all be noted that smoking history and low normal lung coverage are clinically relevant, and can be used to evaluate the benefit:risk ratio of therapy when making treatment decisions for each individual patient.

In addition to the factors outlined above, high pharmacokinetic exposure after a single dose of gefitinib was determined as a potential risk factor for the occurrence of ILD. However, the increased risk of ILD due to high exposure after a single dose is unlikely to be clinically relevant, and it is unlikely that high exposures after a single dose may increase the risk of ILD, while longer-term high exposure does not. The statistically significant impact of high exposure on ILD following single dose administration could be due to the small numbers of patients included in the analysis and/or other factors involved in the occurrence of ILD that have an indirect effect on gefitinib exposure. The odds ratios estimating the association of risk factors to the incidence of ILD showed large confidence intervals (Online Resource, Supplementary Table 1) due to the limited number of patients used in this analysis.

In conclusion, we have for the first time reported the population pharmacokinetics of gefitinib in patients with NSCLC. Exposure to gefitinib at the time of ILD occurrence was significantly elevated, and the increased exposure was due to decreased CL/F, which could be related to ILD-induced AGP elevation. Hence, exposure of gefitinib is unlikely to be a robust predictor and therefore does not warrant dose modification.

## Electronic supplementary material

Below is the link to the electronic supplementary material.

Supplementary material 1 (DOCX 443 KB)
