# Supplementary material for: Gefitinib exposure and occurrence of interstitial lung disease in Japanese patients with non-small-cell lung cancer
Source: Cancer Chemother Pharmacol. 2019 Feb 14;83(5):849–58. doi: 10.1007/s00280-019-03788-4 (PMC6458983; doi:10.1007/s00280-019-03788-4)
Supplement: Supplementary file 1 — Supplementary material 1 (DOCX 443 KB) [file 280_2019_3788_MOESM1_ESM.docx]

**For submission to:** *Cancer Chemotherapy and Pharmacology*

**Online resource**

**Gefitinib exposure and occurrence of interstitial lung disease in Japanese patients with non-small-cell lung cancer**

Toshio Kawata^1^ • Mitsuo Higashimori^1^ • Yohji Itoh^2^ • Helen Tomkinson^3^ • Martin G Johnson^3^ • Weifeng Tang^4^ • Fredrik Nyberg^5^ • Haiyi Jiang^6^ • Yusuke Tanigawara^7^

^1^Clinical Pharmacology & Drug Safety and Metabolism Department, Science Affairs Division, R&D, AstraZeneca K.K., Osaka, Japan
^2^Biometrics Department, Science Affairs Division, R&D, AstraZeneca K.K., Osaka, Japan
^3^Quantitative Clinical Pharmacology, Early Clinical Development, IMED Biotech Unit, AstraZeneca, Cambridge, UK
^4^Quantitative Clinical Pharmacology, Early Clinical Development, IMED Biotech Unit, AstraZeneca, Gaithersburg, MD, USA
^5^Epidemiology, AstraZeneca R&D, Mölndal, Sweden
^6^Immuno-Oncology, Global Medicines Development, AstraZeneca R&D, Gaithersburg, MD, USA
^7^Department of Clinical Pharmacokinetics and Pharmacodynamics, Keio University School of Medicine, Tokyo, Japan

🖂 Professor Yusuke Tanigawara, Ph.D.**,** Department of Clinical Pharmacokinetics and Pharmacodynamics, Keio University School of Medicine, 35 Shinanomachi, Shinjuku-ku, Tokyo 160-8582, Japan; telephone: +81-3-5363-3847; fax: +81-3-5269-4576; e-mail: [tanigawara-yusuke@umin.ac.jp](mailto:tanigawara-yusuke@umin.ac.jp)

# Population pharmacokinetic model development

The population pharmacokinetic analysis was performed using the NONMEM program version V level 1.1 with the PREDPP library and the NM-TRAN pre-processor (GloboMax LLC, Hanover, MD) [1].

Following preliminary modeling to build a base model, log-transformed plasma concentrations were used for the analysis, and a one-compartment model after first-order absorption with lag-time was selected as the structural model. The first-order method was adopted as an approximation in integral of marginal likelihood. The inter-individual variability on pharmacokinetic parameters was described using an exponential error model, and the intra-individual variability on log-transformed plasma concentration was described using an additive error model. The preliminary modeling suggests no covariance among pharmacokinetic parameters was required.

The covariate model was developed based on the forward addition method and the backward deletion method. Continuous covariates such as body weight, age, sex, α_1_-acid glycoprotein (AGP), serum albumin, total protein, aspartate aminotransferase, alanine aminotransferase, alkaline phosphatase, total bilirubin, and creatinine clearance were tested using a power model centered on the mean value.

Categorical covariates included were age group (<65 years or ≥65 years), concomitant use of cytochrome P450 3A4 (CYP3A4) inducers, concomitant use of CYP3A4 inhibitors, and concomitant use of proton pump inhibitors and/or H_2_ antagonists. In addition, interstitial lung disease (ILD) status (i.e. “patients with ILD” vs “patients without ILD”) and ILD period (i.e. “at times other than ILD development” vs “at the time of ILD development”) were tested using a productive multiplicative model with the discrete variable (0 or 1).

χ^2^ tests with one degree of freedom based on the objective function value (OFV) in NONMEM, which corresponds to -2-fold of log-likelihood, were used for developing the covariate model, and the adopted statistically significant levels were p=0.05 (ΔOFV=3.84) and p=0.001 (ΔOFV=10.83) when a covariate was included and excluded from the pharmacokinetic model, respectively.

Goodness-of-fit plots were used for diagnosis of the final model. The robustness of the final model was evaluated by a randomized non-stratified bootstrap technique with 200 resampling datasets [2,3], and model predictability was examined by visual predictive check [4].

Impacts of identified covariates on pharmacokinetic parameters were evaluated using a sensitivity analysis. For continuous covariates, a ratio of pharmacokinetic parameter in patients with deviated values of covariate (mean plus one standard deviation) to patients with mean value of covariate was calculated. For categorical covariates, a ratio of pharmacokinetic parameters between two categories was calculated.

# Population pharmacokinetic model of gefitinib

A one-compartment model after first-order absorption with lag-time (ADVAN2, TRANS2) well described plasma concentration–time profiles. In the final model, statistically significant covariates were AGP, age group, body weight, and concomitant use of CYP3A4 inducers for oral clearance (CL/F), and AGP and body weight for volume of distribution (V/F). In addition, CL/F was significantly lower at the time of ILD diagnosis. The final model and parameter estimates for gefitinib are summarized in Table 2 in the main manuscript. According to the estimated parameters, CL/F and V/F increased with an increase in body weight, and the exponents of AGP terms on CL/F and V/F equations were negative values, suggesting an increase in AGP induced a decrease in CL/F and V/F. Furthermore, it was found that CL/F declined in elderly patients aged ≥65 years or more and in patients using CYP3A4 inducers. All η- and ε-shrinkages [4] were considered acceptable values. The goodness-of-fit plots for the final model showed that model predictions were in reasonable agreement with the observed plasma concentrations (Supplementary Fig. 3).

The 95% bootstrap confidence intervals (CI) suggested that parameter estimates were calculated accurately. Meaningless covariates were not incorporated into the final model, as the 95% bootstrap CI did not include 0 for exponents θ_2_, θ_5_, θ_8_, and θ_9_, and 1 for bases θ_3_, θ_4_, and θ_6_. The visual predictive check demonstrated that observed plasma concentrations were generally contained within 95% prediction intervals (Supplementary Fig. 4).

# References

1. Beal SL, Sheiner LB (1998) NONMEM users guide. University of California.

2. Efron B, Tibshirani RJ (1994) An introduction to the bootstrap. Chapman & Hall, New York.

3. Yafune A, Ishiguro M (1999) Bootstrap approach for constructing confidence intervals for population pharmacokinetic parameters. I: a use of bootstrap standard error. Stat Med 18:581−599.

4. Holford N (2005) The visual predictive check - superiority to standard diagnostic (Rorscharch) plots. <https://www>.page-meeting.org/page/page2005/PAGE2005P105.pdf. Accessed 7/14/2017.

# Tables

**Supplementary Table 1.** Risk factors for acute ILD modelled in the study and estimated odds ratios

| **Risk factor and contrast** | **Analysis 1** | **Analysis 2** | **Analysis 3** | **Analysis 4** |
| --- | --- | --- | --- | --- |
|  | **OR estimate (95% CI)** | **OR estimate (95% CI)** | **OR estimate (95% CI)** | **OR estimate (95% CI)** |
| Age (≥55 vs <55 years) | 1.86 (0.5–6.88) | 1.54 (0.36–6.6) | 2.13 (0.56–8.04) | 1.69 (0.4–7.17) |
| WHO performance status  1 vs 0  2–3 vs 0 | 0.59 (0.2–1.68) 1.75 (0.48–6.33) | 0.55 (0.19–1.62) 1.37 (0.35–5.39) | 0.62 (0.21–1.8) 1.89 (0.52–6.85) | 0.57 (0.19–1.68) 1.34 (0.33–5.39) |
| Smoking history (yes vs no) | 4.46 (1.35–14.76)* | 8.64 (1.74–42.97)* | 4.59 (1.38–15.33)* | 8.15 (1.66–40.08)* |
| Duration of NSCLC  0.5–<1 vs 0.5 years  ≥1 vs <0.5 years | 0.91 (0.33–2.56)  0.37 (0.14–0.95) | 1.89 (0.59–6.07) 0.57 (0.19–1.69) | 0.78 (0.28–2.13) 0.32 (0.12–0.83) | 1.72 (0.53–5.53) 0.59 (0.2–1.75) |
| Pre-existing IP (yes vs no) | 4.01 (0.37–43.22) | 4.49 (0.33–60.44) | 3.56 (0.33–38.74) | 5.21 (0.39–70.35) |
| Normal lung coverage (10%–50% vs 60%–100%) | 3.39 (0.63–18.31) | 9.47 (1.14–78.84)* | 3.56 (0.64–19.85) | 9.4 (1.11–79.48)* |
| Interaction of smoking history and normal lung coverage | 0.6 (0.09–4.12) | 0.14 (0.01–1.52) | 0.56 (0.08–3.96) | 0.17 (0.02–1.89) |
| Interaction of pre-existing IP and normal lung coverage | 0.37 (0.02–6.82) | 0.25 (0.01–6.8) | 0.51 (0.03–9.56) | 0.18 (0.01–4.98) |
| Pharmacokinetic parameter | AUC_0–24_* | AUC_0–24,ss_ | C_max_* | C_max,ss_ |
| Quartile 2 vs Quartile 1 | 0.68 (0.2–2.32) | 1.2 (0.27–5.32) | 0.51 (0.14–1.8) | 0.69 (0.16–3.02) |
| Quartile 3 vs Quartile 1 | 0.62 (0.18–2.12) | 1.71 (0.42–6.94) | 0.5 (0.14–1.72) | 1.72 (0.47–6.27) |
| Quartile 4 vs Quartile 1 | 3.35 (1.14–9.88) | 2.09 (0.5–8.74) | 3.03 (1–9.17) | 1.07 (0.27–4.29) |

Unless stated otherwise, data are statistically non-significant (p>=0.05).
*p<0.05 (significant).
AUC_0–24,_ area under the plasma concentration–time curve from 0–24 hours after a single dose; AUC_0–24,ss,_ area under the plasma concentration–time curve from 0–24 hours at steady-state; CI, confidence interval; C_max_, maximum plasma concentration after a single dose; C_max,ss_, maximum plasma concentration at steady-state; ILD, interstitial lung disease; IP, interstitial pneumonia; NSCLC, non-small-cell lung cancer; OR, odds ratio; Q, quartile; WHO, World Health Organization.

# Figures

**Supplementary Fig. 1** Schematic illustration of study groups in the cohort and nested case-control study and identification of patients for population pharmacokinetic (PPK) analysis

| Cohort | All patients with advanced/recurrent non-small-cell lung cancer (NSCLC) participating in this post-marketing clinical study |
| --- | --- |
| Gefitinib-treated patients | Patients in the cohort with advanced/recurrent NSCLC who received gefitinib |
| Case-control study | Patients enrolled in the case-control study; all consenting patients with interstitial lung disease (ILD) designated as **“cases”,** with approximately 4-times as many consenting patients without ILD randomly selected from the cohort and designated as **“controls”** |

**Supplementary Fig. 2** Sampling of cases and 4 controls per case from the cohort

**Supplementary Fig. 3** Diagnosis plots for the final population pharmacokinetic model of gefitinib. Scatter plots show: a) the goodness-of-fit between observed concentrations and population-predicted concentrations; b) the goodness-of-fit between observed concentrations and individual-predicted concentrations; c) the goodness-of-fit between population-predicted concentrations and weighted residuals; and d) the goodness-of-fit between individual-predicted concentrations and individual-weighted residuals. The solid line represents the identical line, and the dotted line represents the smoothing curve by locally weighted scatterplot smoothing (LOWESS)

**a)**


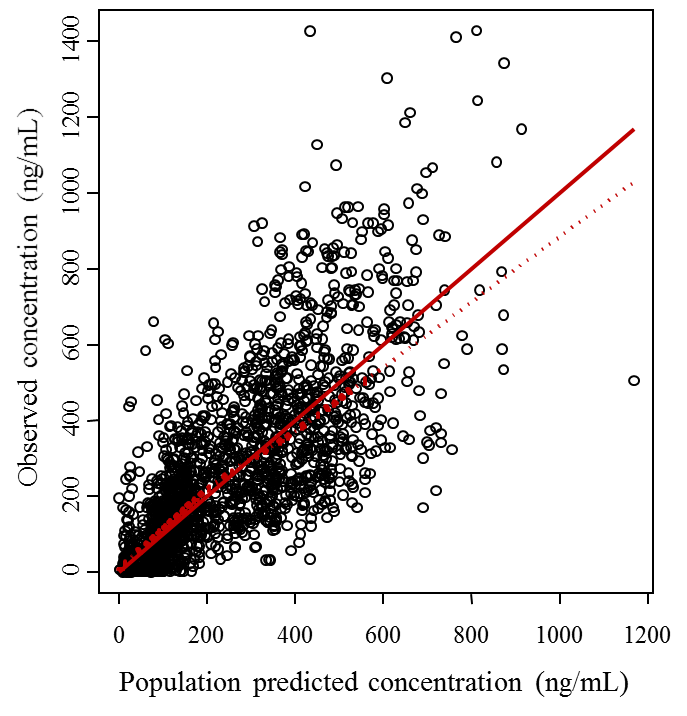


**b)**


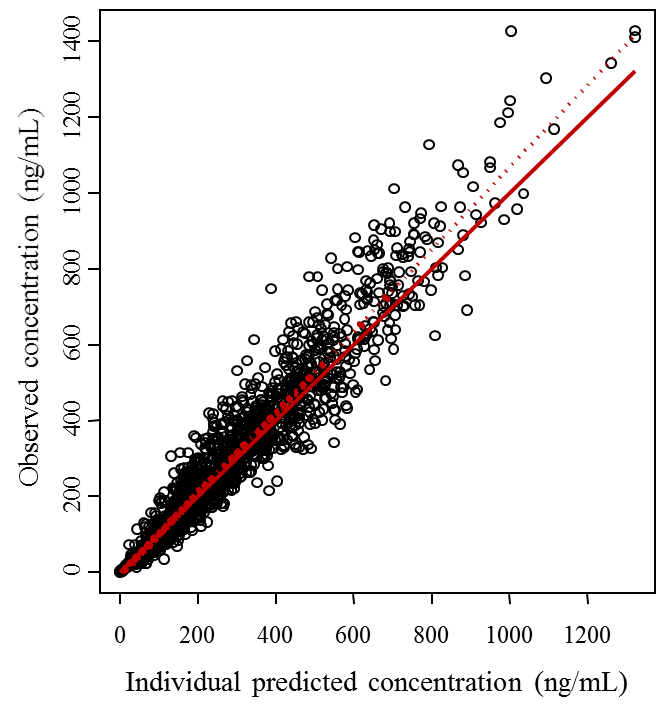


**c)**


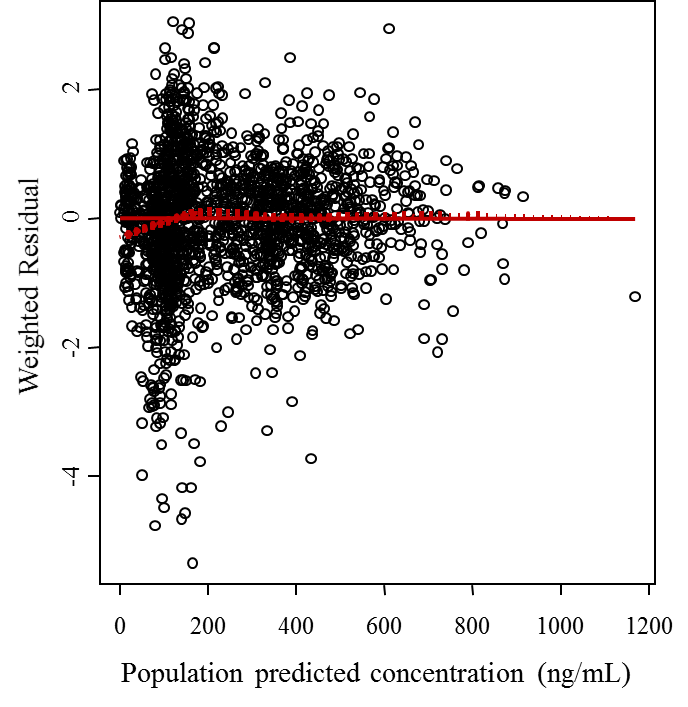


**d)**


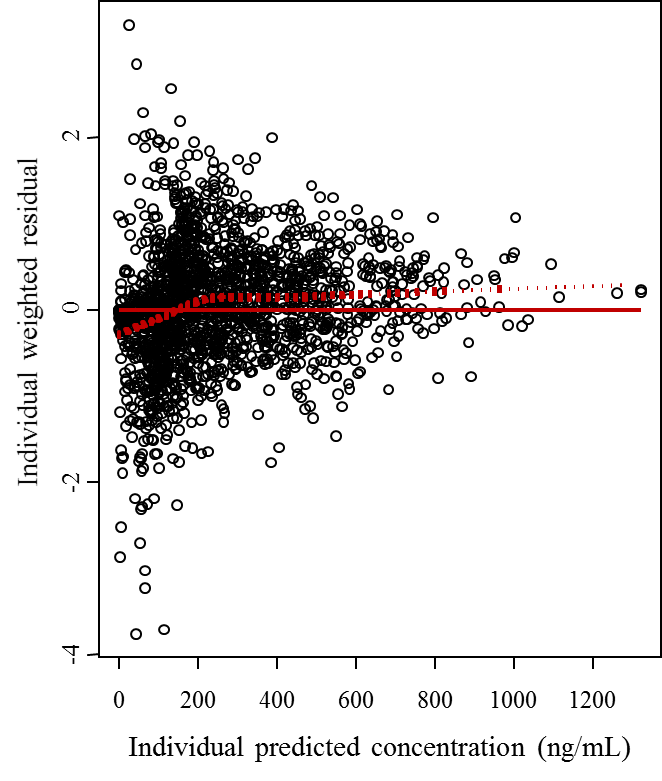


**Supplementary Fig. 4** Visual predictive checks of steady-state plasma concentration of gefitinib: a) at times other than ILD development; and b) at the time of ILD development. The solid line is the median of predicted plasma concentrations, and the dotted lines are the 2.5^th^ and 97.5^th^ percentiles of the predicted plasma concentrations. Dots represent measured concentrations directly after dosing and approximately 24h after dosing. ILD, interstitial lung disease

**a)**


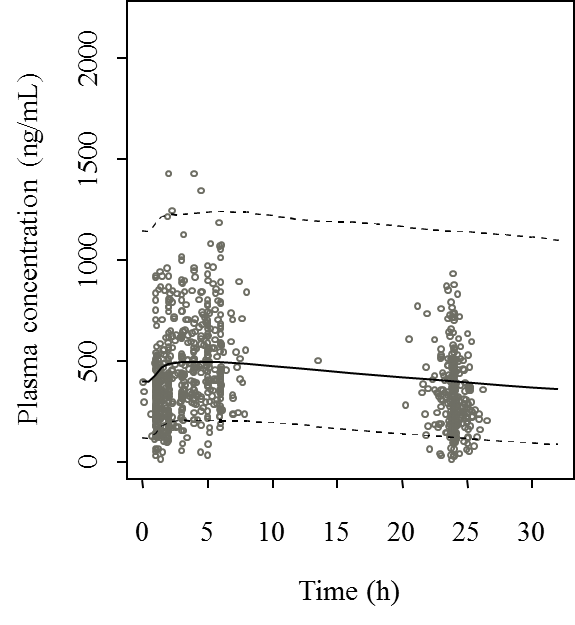


**b)**


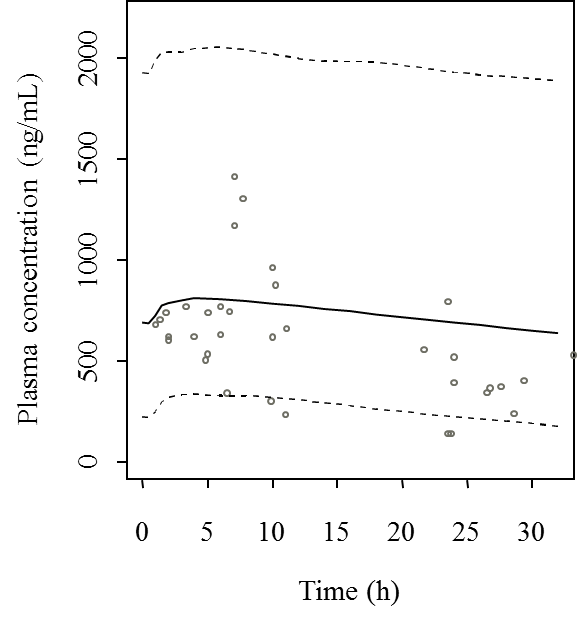


# Hospitals and Principal Investigators contributing to the study

National Hospital Organization Hokkaido Cancer Centre (Hiroshi Isobe), Hokkaido University Hospital (Koichi Yamazaki), National Hospital Organization Dohoku National Hospital (Yuka Fujita), Tohoku University Hospital (Akira Inoue), Sendai Kousei Hospital (Shunichi Sugawara), National Cancer Centre Hospital East (Yutaka Nishiwaki), Nippon Medical School Chiba Hokusoh Hospital (Yasushi Ono), Tokyo Medical University Hospital (Masahiro Tsuboi), Nippon Medical School Hospital (Tetsuya Okano), Toho University Omori Medical Centre (Nobuyuki Hamanaka), Toranomon Hospital (Kunihiko Yoshimura), National Hospital Organization Tokyo Hospital (Atsuhisa Tamura), Juntendo University Hospital (Kazuhisa Takahashi), Kyorin University Hospital (Tomoyuki Goya), Tokai University Hospital (Kenji Eguchi), Kitasato University School of Medicine (Noriyuki Masuda), Kanagawa Cardiovascular and Respiratory Centre (Takashi Ogura), Niigata Cancer Centre Hospital (Akira Yokoyama), National Nishi-Niigata Central Hospital (Hiromi Miyao), Toyama University Hospital (Muneharu Maruyama), Kanazawa University Hospital (Kazuo Kasahara), Aichi Hospital, Aichi Cancer Centre (Hiroshi Saito), National Hospital Organization Nagoya Medical Centre (Hideo Saka), Fujita Health University Hospital (Hiroki Sakakibara), Nagoya Ekisaikai Hospital (Masashi Yamamoto), Shiga University of Medical Science Hospital (Noriaki Tezuka), Kyoto Katsura Hospital (Takeshi Hanawa), National Hospital Organization Kyoto Medical Centre (Yoshiyuki Sasaki), Rinku General Medical Centre Municipal Izumisano Hospital (Hisao Uejima), Kinki University, School of Medicine (Kazuhiko Nakagawa), National Hospital Organization Kinki-chuo Chest Medical Centre (Masaaki Kawahara), Osaka City General Hospital (Koji Takeda), Osaka City General Hospital (Hirohito Tada), Osaka City University Hospital (Shinzoh Kudoh), Osaka Prefectural Medical Centre for Respiratory and Allergic Diseases (Kaoru Matsui), Osaka Police Hospital (Kiyoshi Komuta), Toneyama National Hospital (Soichiro Yokota), Kobe City General Hospital (Keisuke Tomii), Hyogo Medical Centre for Adults (Shunichi Negoro), Kobe University Hospital (Yoshihiro Nishimura), Institute of Biomedical Research and Innovation (Nobuyuki Katakami), Tenri Hospital (Yoshio Taguchi), Okayama University Medical and Dental School Hospital (Katsuyuki Kiura), Hiroshima City Hospital (Hidetaka Sumiyoshi), Hiroshima City Hospital (Noritomo Senoo), National Hospital Organization Shikoku Cancer Centre (Tetsu Shinkai), National Hospital Organization Kyushu Cancer Centre (Yukito Ichinose), Fukuoka National Hospital (Akira Motohiro), University of Occupational and Environmental Health (Masamitsu Kido), University of Occupational and Environmental Health (Kenji Sugio), National Hospital Organization Nagasaki Medical Centre (Akitoshi Kinoshita), Kumamoto University Hospital (Mitsuhiro Matsumoto), Kumamoto-Chuo Hospital (Sunao Ushijima), Okinawa National Hospital (Mutsuo Kuba).
